# Chronic Inflammatory Demyelinating Polyneuropathy in a Patient With Autoimmune Autonomic Ganglionopathy: A Case Report

**DOI:** 10.7759/cureus.106422

**Published:** 2026-04-04

**Authors:** Emily N Taylor, Jason E Hansen, Justin White

**Affiliations:** 1 Department of Medicine, University of New England College of Osteopathic Medicine, Portland, USA; 2 Department of Hospital Medicine, Northern Light AR Gould Hospital, Presque Isle, USA

**Keywords:** autoimmune autonomic ganglionopathy, autoimmune illness, autoimmune inflammatory demyelinating polyneuropathy, chronic inflammatory demyelinating polyneuropathy (cidp), guillain-barre syndrome (gbs)

## Abstract

This case describes a 61-year-old woman with autoimmune autonomic ganglionopathy (AAG) on long-term intravenous immunoglobulin (IVIG) who developed progressive weakness and sensory loss, ultimately diagnosed as chronic inflammatory demyelinating polyneuropathy (CIDP). Initially evaluated for Guillain-Barré syndrome, cerebrospinal fluid analysis revealed albuminocytologic dissociation without central lesions on MRI. Despite IVIG therapy, symptoms worsened, and electrodiagnostic testing demonstrated demyelination with conduction block and secondary axonal loss, confirming CIDP. Subsequent plasma exchange therapy led to gradual improvement in strength, reflexes, and ambulation while autonomic function remained stable. The coexistence of CIDP and AAG is exceedingly rare and emphasizes the potential for shared autoimmune mechanisms within the peripheral nervous system. This case highlights the importance of recognizing overlapping autoimmune neuropathies, particularly when new or progressive symptoms arise despite appropriate therapy and adapting immunomodulatory treatment accordingly to optimize long-term outcomes.

## Introduction

Autoimmune autonomic ganglionopathy (AAG) is a rare antibody-mediated disorder that disrupts autonomic nervous system function. Its exact incidence is unknown, though only about 100 cases are diagnosed each year [1]. The disease is the result of antibodies directed against the ganglionic acetylcholine receptor (gAChR), a nicotinic receptor present at synapses within autonomic ganglia. The binding of these autoantibodies disrupts synaptic communication between pre- and postganglionic autonomic neurons, leading to impaired signaling in both sympathetic and parasympathetic pathways. As a result, AAG causes widespread autonomic failure, manifesting as orthostatic hypotension, gastrointestinal dysmotility, pupillary dysfunction, and anhidrosis, while typically sparing motor and sensory function [2]. Treatment classically involves routine infusions of intravenous immunoglobulin (IVIG). Refractory cases may require plasmapheresis or high-dose immunosuppression.

Chronic inflammatory demyelinating polyneuropathy (CIDP) is a rare, immune-mediated disorder of the peripheral nervous system. Unlike AAG, CIDP is characterized by progressive or relapsing sensorimotor deficits lasting at least eight weeks [3]. It is considered the chronic counterpart of acute inflammatory demyelinating polyradiculoneuropathy (AIDP), the most common variant of Guillain-Barré syndrome (GBS). CIDP affects about two people per 100,000 people each year [4]. The disease is known to be associated with illnesses such as systemic lupus erythematosus, HIV, and hepatitis. Regardless of the trigger, CIDP leads to complement-mediated, T-cell-mediated, and humoral-mediated destruction of myelin in the peripheral nervous system, leading to demyelination. The specific antigen interaction leading to CIDP is unknown. Specific autoantibodies are known to be associated with this disease. However, these tests have a low sensitivity as antibodies may only be positive in up to 18% [2]. The specific proteins targeted in CIDP include neurofascin and contactin-associated protein 1, both of which help maintain attachment of myelin to peripheral nerves [3]. Diagnosis relies on the clinical course, electrodiagnostic evidence of demyelination, and supportive findings such as elevated cerebrospinal fluid (CSF) protein without inflammatory cells [5]. Although CIDP is well described, its overlap with other autoimmune neuropathies remains uncommon and diagnostically challenging.

Given the rarity of both disorders, overlapping cases are extremely uncommon and have not been well documented in the literature. Overlapping immunopathology can confuse the clinical presentation and complicate diagnosis. The simultaneous occurrence of AAG and CIDP presents unique diagnostic and management challenges that are important for clinicians to recognize.

We present a case of a 61-year-old female with known AAG, receiving chronic IVIG, who developed progressive weakness and sensory changes ultimately diagnosed as CIDP. This case highlights the challenge of recognizing overlapping autoimmune neuropathies in patients on immunotherapy, where baseline treatment may mask or alter symptom presentation. It emphasizes that new neurologic symptoms in patients with established autoimmune disease should not automatically be attributed to the known condition, illustrating a critical knowledge gap in recognizing and managing autoimmune neuropathies. Although the diagnostic workup can be complex, careful evaluation is essential to detect concurrent disorders and guide appropriate management [2].

## Case presentation

A 61-year-old female with an established five-year history of AAG, associated with high titers of gAChR, presented to the emergency department with new-onset paresthesia in her fingers and toes, which had started just under one month prior without an inciting incident. She had no other significant past medical history. At the time of her AAG diagnosis, her anti-gAChR antibody titer was seventy-four times the normal level, at 1.47 mmol/L (reference <0.02 mmol/L). The most recent titer, obtained approximately one year before presentation, was 0.71 mmol/L. She was typically well-managed with IVIG 70 g every three weeks, with the most recent dose administered two weeks before presentation. Her baseline AAG manifestations included chronic orthostatic tachycardia, constipation, hyperhidrosis, and urinary retention. At baseline, she had no skin or nail changes. She had no previous history of dysthesias or weakness before this presentation.

She was determined to be clinically stable in the emergency department and was discharged, with outpatient follow-up with primary care scheduled in one week. She presented again six days later with progressing bilateral lower extremity sensory loss and new weakness, primarily in her lower extremities, that required elbow crutches for support. These new and progressive neurological findings prompted admission for further investigations to determine the underlying cause. 

**Figure 1:**
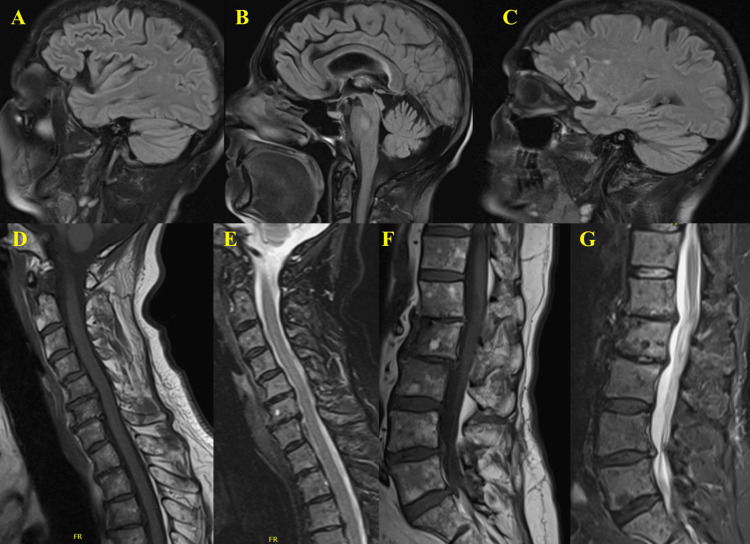
MRI of the brain, cervical spine, and lumbar spine. Brain MRI (images A-C) demonstrates no acute infarct, intracranial hemorrhage, or mass effect, with white matter changes consistent with chronic small vessel ischemia and no evidence of demyelination or abnormal enhancement, with image A representing the right side, image B the midline, and image C the left side; cervical spine MRI demonstrates moderate multilevel spondylosis with mild canal stenosis and no cord signal abnormality or abnormal cord enhancement, with image D representing a T1-weighted sequence and image E representing a T2-weighted sequence; and lumbar spine MRI demonstrates no abnormal enhancement or critical canal stenosis, with image F representing a T1-weighted sequence and image G representing a T2-weighted sequence.

On the day of admission, the patient was alert and oriented with intact cranial nerves. She had profound bilateral lower extremity weakness with near-complete inability to flex her hips or dorsiflex her feet, impairing ambulation. Despite the severity of weakness, she had not noticed any associated muscle wasting. She also denied neuropathic pain or symptoms suggestive of small fiber involvement. She had absent or barely elicitable knee reflexes and sensory loss up to the knees. The initial differential diagnosis included transverse myelitis, GBS, and multiple sclerosis (MS), to encompass both central and peripheral causes of subacute weakness and sensory changes. Diagnostic CT of the lumbar spine was performed to evaluate for structural or compressive causes rapidly, and showed no acute changes. MRI of the brain, cervical spine, and lumbar spine was performed for a more sensitive assessment of the spinal cord and soft tissues. However, no abnormalities were identified on MRI, making MS and transverse myelitis less likely (Figure 1). Additionally, the absence of a distinct sensory level, hyperreflexia, or bowel and bladder dysfunction further reduced the likelihood of transverse myelitis. The absence of upper motor neuron signs pointed toward a peripheral rather than central etiology, supporting GBS as the leading differential. Overall, the imaging results combined with the clinical findings reinforced a peripheral demyelinating process as the cause of the patient's symptoms.

A lumbar puncture demonstrated albuminocytologic dissociation, with elevated CSF protein, a normal CSF white blood cell (WBC) count, and absent oligoclonal bands (Table 1). Extensive CSF testing, including autoimmune, paraneoplastic, and VDRL panels, was negative.

**Table 1:** Cerebrospinal fluid studies. CSF studies demonstrating a normal WBC count and elevated CSF protein. CSF, cerebrospinal fluid; WBC, white blood cell

CSF study	Patients value	Reference value
CSF WBC count	0 cells/µL	0 cells/µL
CSF protein	82.9 mg/dL	15.0-45.0 mg/dL
CSF oligoclonal bands	Absent	Absent
CSF T. Pallidum (VDRL) Screen	Nonreactive	Nonreactive

Neurology was consulted, and she was started on the first-line treatment of a five-day course of IVIG for suspicion of GBS. After completion, she demonstrated some improvement in movement and sensation, but had not yet returned to her baseline. She was discharged home with outpatient neurology follow-up. Throughout her stay, she received five days of IVIG. She regained strength in her legs and was able to ambulate with the assistance of a rolling walker, prompting discharge with a follow-up with neurology. She then presented again a week later with progressing bilateral lower extremity sensory loss and worsening weakness that required elbow crutches for support. These new findings prompted reevaluation and admission. 

At the outpatient neurology follow-up eight days later, the patient reported being unable to maintain her usual ADLs after discharge. She had been receiving outpatient physical and occupational therapy but remained largely dependent on her husband, requiring a wheelchair due to worsening weakness and paresthesia. The patient did not experience any red flag neurologic symptoms or symptoms consistent with disease progression. On examination in the office, an attempt to stand from her wheelchair revealed profound proximal lower extremity weakness and inability to support herself independently. Upper extremity strength remained intact. Sensory testing showed reduced pinprick sensation extending from the bilateral lower extremities to the torso, with preservation of facial sensation. Vibration sense was preserved at the toes.

Based on the patient's clinical picture, eight weeks of deterioration with lack of improvement from IVIG therapy, neurology recommended hospital admission. Confirmatory testing was recommended, as the symptom timeline was highly suspicious for CIDP rather than AIDP. 

The patient was readmitted for electromyography (EMG) and nerve conduction studies (Tables 2-3). Testing of the right upper extremity and bilateral lower extremities showed prolonged distal latencies, reduced amplitudes, and slowed or blocked conduction in multiple sensory and motor nerves. F-waves were absent or prolonged, indicating diminished muscle response to nerve stimulation in bilateral lower extremity nerves. Needle EMG demonstrated denervation in several distal and proximal muscles, indicating secondary axonal loss consistent with demyelination leading to conduction block. This diffuse demyelination and conduction block pattern over a duration longer than eight weeks suggests a chronic or relapsing process, such as CIDP, rather than a monophasic process such as GBS.

**Table 2:** Nerve conduction studies. Results showing upper and lower extremity nerves with prolonged distal latencies, reduced amplitudes, and slowed/blocked conduction. F-waves were absent or prolonged, indicating diminished muscle response to nerve stimulation in bilateral lower extremity nerves.

Left or right	Nerve	Motor or sensory	Distal stimulus distance (cm)	Latency (ms)		Amplitude		Conduction velocity (m/s)	
				Recorded	Normal	Recorded	Normal	Recorded	Normal
R	Sural	S	14	3.3	<4.3	4.1	>5 uV	-	-
R	Peroneal	S	14	5.2	<4.3	2.2	>8 uV	-	-
R	Peroneal	M	8	7.4	<5.6	0.4/0.1	>2 mV	23	>40
R	Tibial	M	8	10.2	<6.2	2.6/0.9	>2 mV	35	>40
L	Sural	S	14	4.0	<4.3	1.1	>5 uV	-	-
L	Peroneal	S	14	5.4	<4.3	1.6	>8 uV	-	-
L	Peroneal	M	8	9.4	<5.6	2.0/0.9	>2 mV	32	>40
L	Tibial	M	8	8.9	<6.2	1.9 / 1.2	>2 mV	37	>40
R	Median	S	14	6.3	<3.8	2.3	>20 uV	-	-
R	Ulnar	S	14	4.3	<3.7	19.2	>10 uV	-	-
R	Median	M	8	7.9	<4.3	5.4/4.5	>4 mV	49	>49
R	Ulnar	M	8	3.9	<4.0	4.3/2.5	>5 mV	40	>49
R	Radial	S	10	2.4	<2.7	18.3	>13 uV	-	-

**Table 3:** Electromyography studies Results suggesting denervation in several muscles, indicating secondary axonal loss consistent with demyelination and conduction block.

Left or right	Muscle	Innervation	Spontaneous activity		Voluntary activity				
			Positive waves	Fibrillations	Amplitude	Duration	Polyphasicity	Recruitment	Interference pattern
R	Vastus Medialis	L2-4 Femoral	1-2+	1-2+	N	N	N	N	N
R	Tibialis anterior	L4-5 Deep Peroneal	2+	2+	N	N	N	N	N
R	Peroneus Longus	L5-S1 Sup. Peroneal	2+	2+	N	N	N	N	N
R	Medial Hamstring	L4-S1 Sciatic	1-2+	1-2+	N	N	N	N	N
R	Medial Gastrocnemius	S1-S2 Tibial	2-3+	2-3+	N	N	N	N	N
L	Vastus Medialis	L2-4 Femoral	1-2+	1-2+	N	N	N	N	N
L	Anterior Tibialis	L4-5 Deep Peroneal	2+	2+	N	N	N	N	N
L	Peroneus Longus	L5-S1 Sup. Peroneal	2+	2+	N	N	N	N	N
L	Medial Hamstring	L4-S1 Sciatic	1-2+	1-2+	N	N	N	N	N
L	Medial Gastrocnemius	S1-S2 Tibial	2-3+	2-3+	N	N	N	N	N
R	Deltoid	C5-6 Axially	0	0	N	N	N	N	N
R	Biceps	C5-6 Musculocutaneous	0	0	N	N	N	N	N
R	Flexor Carpi Radialis	C6-7 Median	0	0	N	N	N	N	N
R	Triceps	C7-8 Radial	0	0	N	N	N	N	N
R	1st Dorsal Interosseus	C8-T1 Ulnar	0	0	N	N	N	N	N

Based on these electrodiagnostic findings, combined with the clinical presentation, the patient met diagnostic criteria for CIDP. In accordance with the European Academy of Neurology/Peripheral Nerve Society guidelines, nerve biopsy was not pursued, as it is not recommended as a routine diagnostic procedure and is reserved for cases in which CIDP cannot be confirmed through electrodiagnostic evaluation.

The patient was subsequently started on five rounds of plasma exchange (PLEX) therapy over 10 days. CIDP-specific antibody testing was not performed, as the diagnosis had already been established based on clinical presentation and electrodiagnostic studies, and serologic testing was not required for diagnosis. 

After the first and second PLEX therapy, the patient reported transient worsening of numbness in her hands and feet and persistent weakness in both upper and lower extremities, remaining unable to stand. Sensation to light touch and pinprick remained diminished, and deep tendon reflexes were absent bilaterally. Examination after the third treatment showed improvement in dorsiflexion, plantar flexion, and toe movements bilaterally. Despite persistent numbness in her feet and hands, she was able to ambulate short distances with a walker, exhibiting a sliding gait due to limited foot dorsiflexion. She continued to make small improvements in motor function following the fourth treatment. After the final PLEX, she reported marked improvement, including greater foot lift and assisted stair climbing. Both the paresthesia and reflexes improved markedly. Her autonomic function remained stable at baseline. Overall, the patient demonstrated a strong clinical response to PLEX therapy, further supporting an immune-mediated demyelinating process consistent with CIDP.

Overall, the patient demonstrated gradual but meaningful improvement in strength and sensation over the course of PLEX therapy, permitting discharge in a stable condition. Plans were made for placement of a long-term plasmapheresis catheter for maintenance therapy consisting of three PLEXs every four weeks in the outpatient setting.

## Discussion

This patient’s presentation of progressive, ascending weakness with sensory changes initially raised concern for GBS. However, the gradual evolution of symptoms over eight weeks, coupled with electrodiagnostic evidence of conduction block and axonal involvement, was more consistent with CIDP. Both CIDP and GBS involve immune-mediated demyelination of peripheral nerves, but they differ in rate of progression and chronicity [3]. GBS typically peaks within four weeks and often lacks early conduction block or axonal degeneration, whereas CIDP progresses beyond eight weeks or follows a relapsing course with segmental demyelination and secondary axonal loss [5]. Recognizing these temporal and electrophysiologic distinctions is essential, since CIDP requires long-term immunomodulatory therapy rather than the short-term management typical of GBS.

An especially intriguing aspect of this case is the coexistence of AAG and CIDP. AAG is mediated by antibodies targeting the gAChR, resulting in symptoms such as orthostatic hypotension, constipation, and urinary retention without motor or sensory involvement. CIDP is associated with autoantibodies against myelin proteins and gangliosides and primarily affects peripheral motor and sensory nerves while sparing autonomic function. Although each disorder is characterized by a distinct immunopathology, their coexistence may reflect a more general dysregulation of the immune system. The presence of both disorders is rare but may occur through mechanisms that disrupt self-tolerance and lead to the formation of autoantibodies targeting different components of the peripheral nervous system [2,6]. 

There are no published case reports describing concurrent CIDP and AAG. However, overlap between CIDP and other antibody-mediated neurologic autoimmune disorders has been described, including rare reports of CIDP occurring concurrently with myasthenia gravis. In one case, a middle-aged patient was diagnosed with CIDP based on progressive sensorimotor demyelinating polyneuropathy, albuminocytologic dissociation, and improvement with IVIG. He later developed fluctuating bulbar weakness and fatigability with electrophysiologic evidence of neuromuscular junction dysfunction and positive acetylcholine receptor antibodies, confirming a diagnosis of MG [7]. The significance of overlapping neurologic autoimmune disorders is unclear, highlighting the need for further investigation. 

Autoimmune disorders are known to develop with increased frequency in patients with a previous diagnosis of autoimmunity, with up to 25% of patients developing additional autoimmune diseases, suggesting a shared immune dysregulation [8]. The pathogenesis of autoimmune disorders is still not completely understood, but it is thought to result from environmental triggers in genetically susceptible individuals. Proposed mechanisms for concurrent autoimmune neurologic diseases include antigen-driven, major histocompatibility complex-restricted T-cell responses with secondary B-cell activation, production of pathogenic autoantibodies, and impaired regulatory T-cell function, resulting in a loss of immune tolerance across multiple tissues [7]. The presence of both syndromes may indicate a heightened immune activity and possibly a more refractory disease course.

In this patient, consistent IVIG therapy for AAG may have temporarily suppressed the regulatory immune response, yet CIDP still developed. Continued clinical deterioration despite regular IVIG therapy led to the identification of a second neuropathic process. While IVIG is considered first-line therapy for CIDP, approximately twenty to thirty percent of patients are refractory [9]. Patients with distal CIDP are more often refractory to IVIG, but are shown to respond well to other therapies like plasmapheresis and corticosteroids [6,9]. The mechanisms underlying IVIG resistance are not fully understood. It is documented that refractory patients often present with more severe neurological impairment and peripheral nerve damage [9]. This case highlights the importance of considering a second autoimmune neuropathy with new or progressive symptoms despite treatment. Given ongoing deterioration despite IVIG, PLEX was initiated, leading to gradual improvement in motor function and reduction of paresthesia. 

## Conclusions

This case emphasizes the importance of distinguishing CIDP from GBS and central demyelinating disorders, as management strategies, treatment duration, and prognosis differ significantly between these two conditions. It also illustrates the rare clinical overlap between autonomic and somatic autoimmune neuropathies, suggesting the possibility of a shared autoimmune dysregulation within the peripheral nervous system. Most importantly, new or evolving neurologic symptoms in patients with established autoimmune neurologic disease should prompt diagnostic reassessment rather than being attributed to progression of the original disorder. In this patient, the development of new sensorimotor deficits, lack of sustained response to IVIG, and symptom progression beyond eight weeks signaled the presence of a second autoimmune neuropathy. Early recognition of overlapping immune-mediated processes, supported by timely diagnostic evaluation, is critical to guide individualized immunotherapy and optimize patient outcomes.
